# Supplementary figures and images for: Interpreting coronary artery disease GWAS results: A functional genomics approach assessing biological significance
Source: PLoS One. 2022 Feb 22;17(2):e0244904. doi: 10.1371/journal.pone.0244904 (PMC8863290; doi:10.1371/journal.pone.0244904)

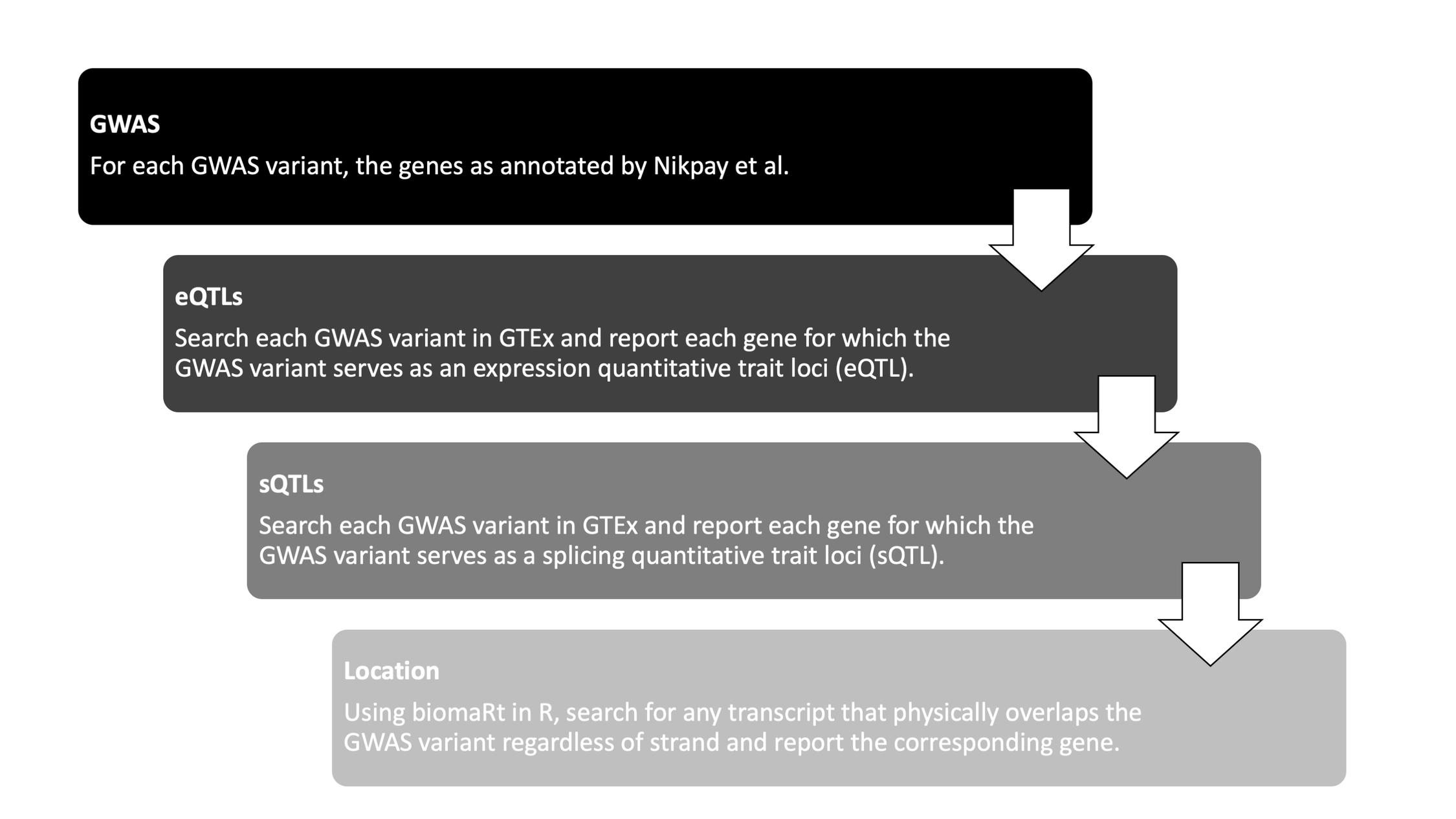

Supplement: S1 Fig — Flowchart portraying process of expanding candidate gene list from 75 to 245 using eQTL, sQTL, and physical position. (TIF) [file pone.0244904.s001.tif]

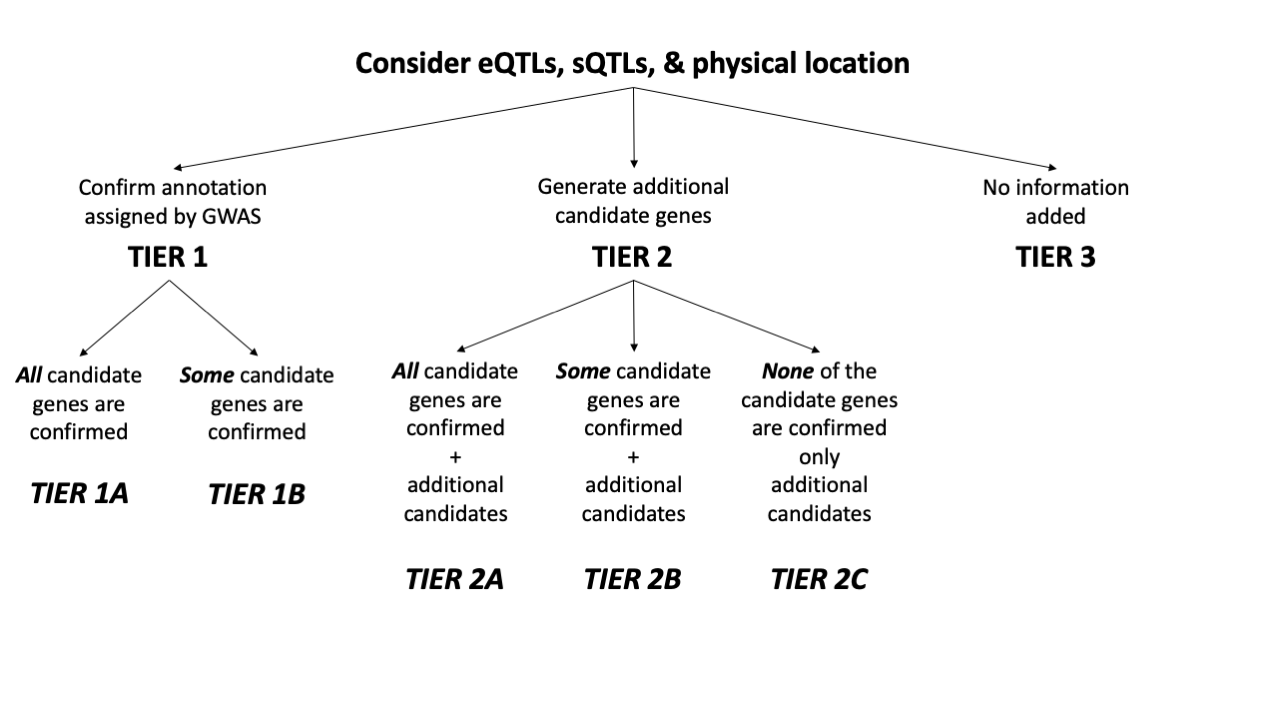

Supplement: S2 Fig — Flowchart portraying process of assigning tiers to CAD GWAS loci. (TIF) [file pone.0244904.s002.tif]

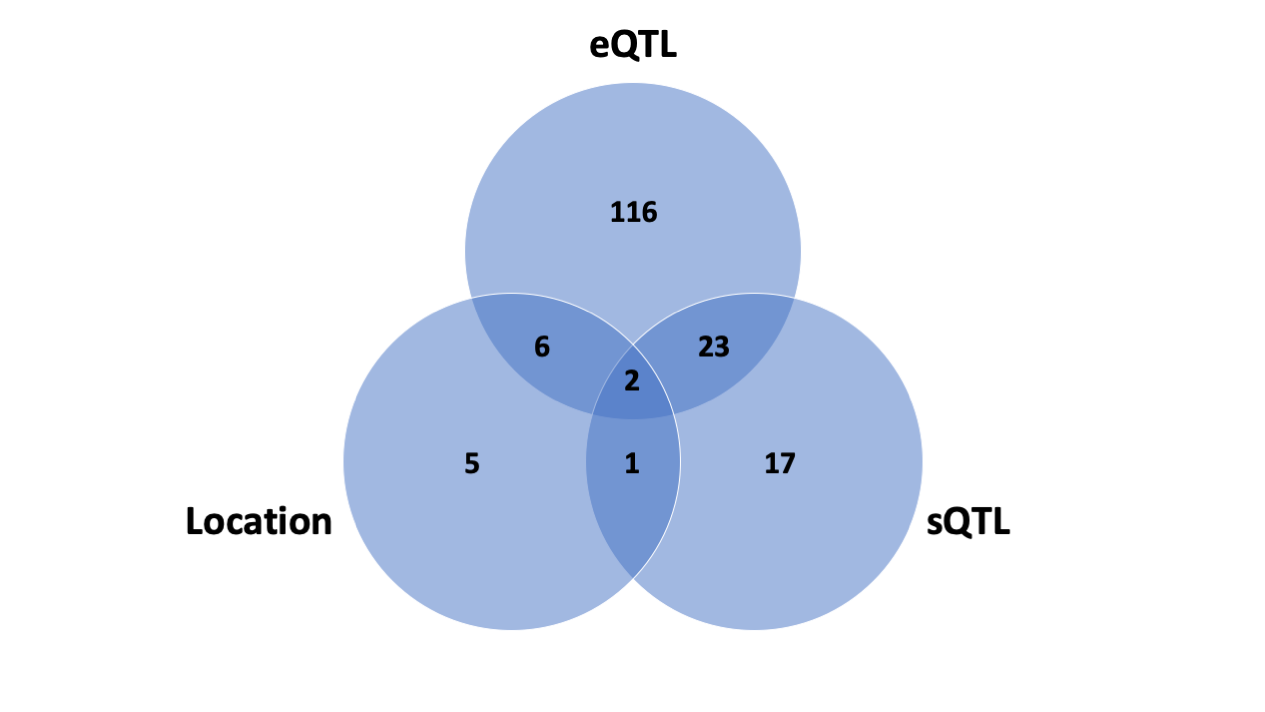

Supplement: S3 Fig — Venn diagram showing overlap in candidate genes derived from eQTL, sQTL, and position-based re-prioritization. (TIF) [file pone.0244904.s003.tif]

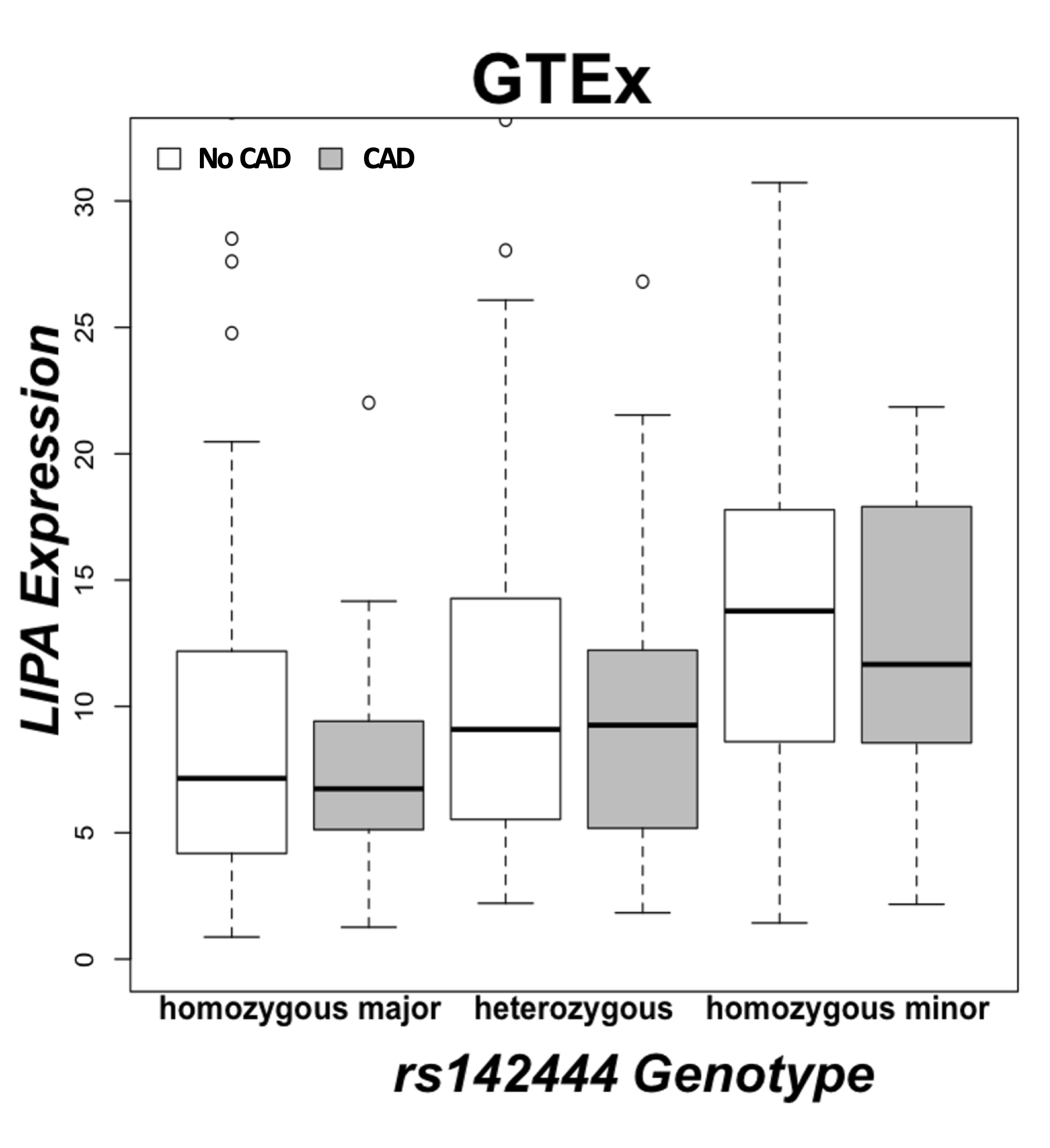

Supplement: S4 Fig — Comparison of LIPA expression in GTEx for those with and without heart disease based on rs142444 genotype. LIPA exhibits higher expression in those without heart disease only in the homozygous minor group (p value = 0.22). (TIF) [file pone.0244904.s004.tif]

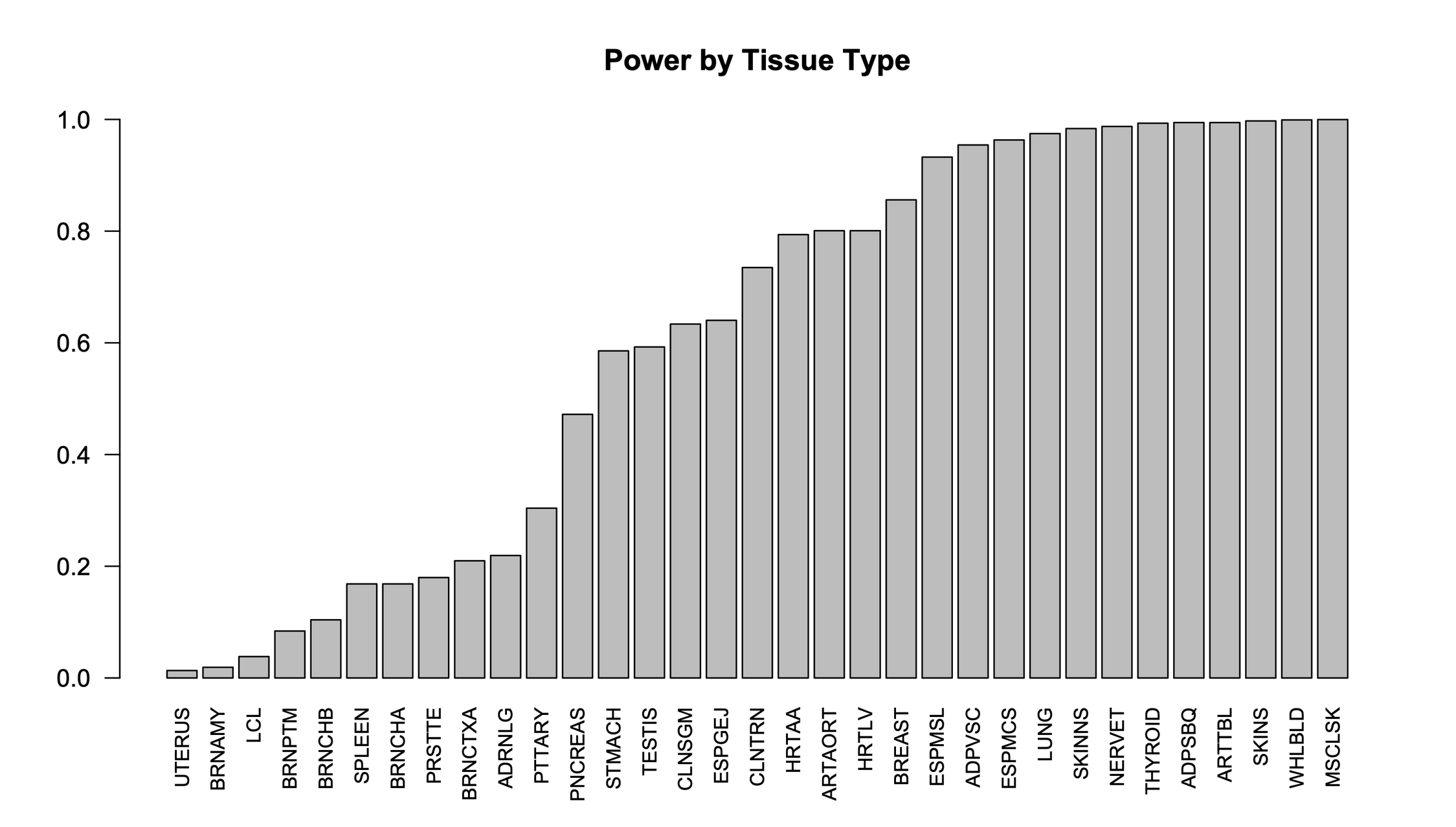

Supplement: S5 Fig — Barplot displays power to detect a hypothetical LIPA eQTL with minor allele frequency 0.05 and effect size 40% (i.e. no minor alleles is 20% less than the median tissue specific gene expression and two minor alleles is 20% greater than the median expression) across different tissue types. About half of the tissues have greater than 80% power to detect such a variant. (TIF) [file pone.0244904.s005.tif]

CAD (Nikpay et. al)

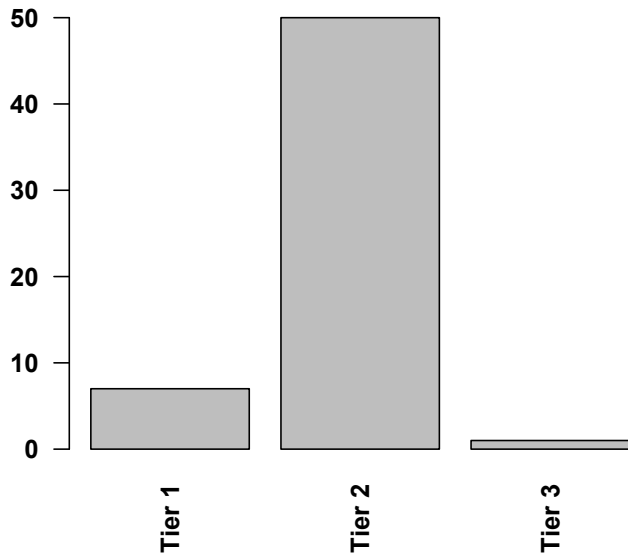

Insulin Resistance (Lotta et. al)

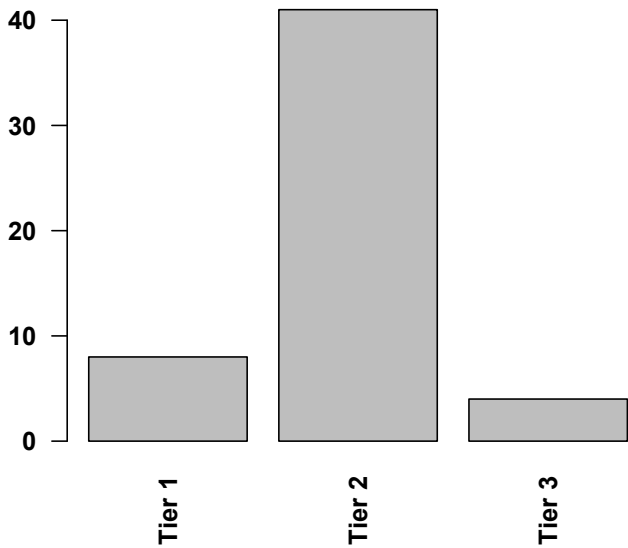

Blood Pressure (Evangelou et. al)

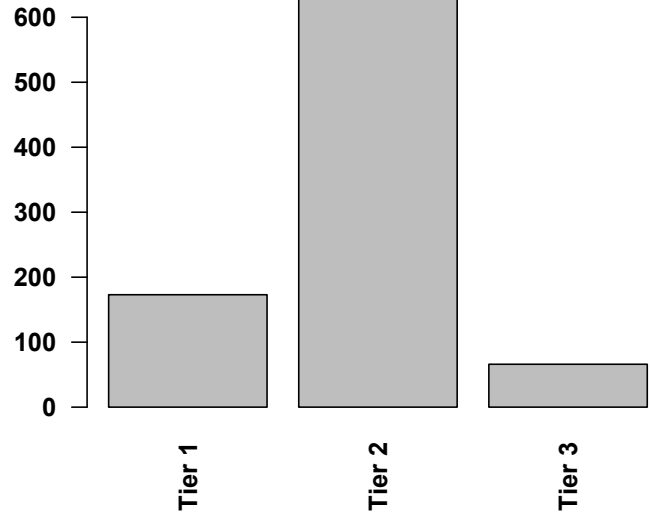

# TIER 1

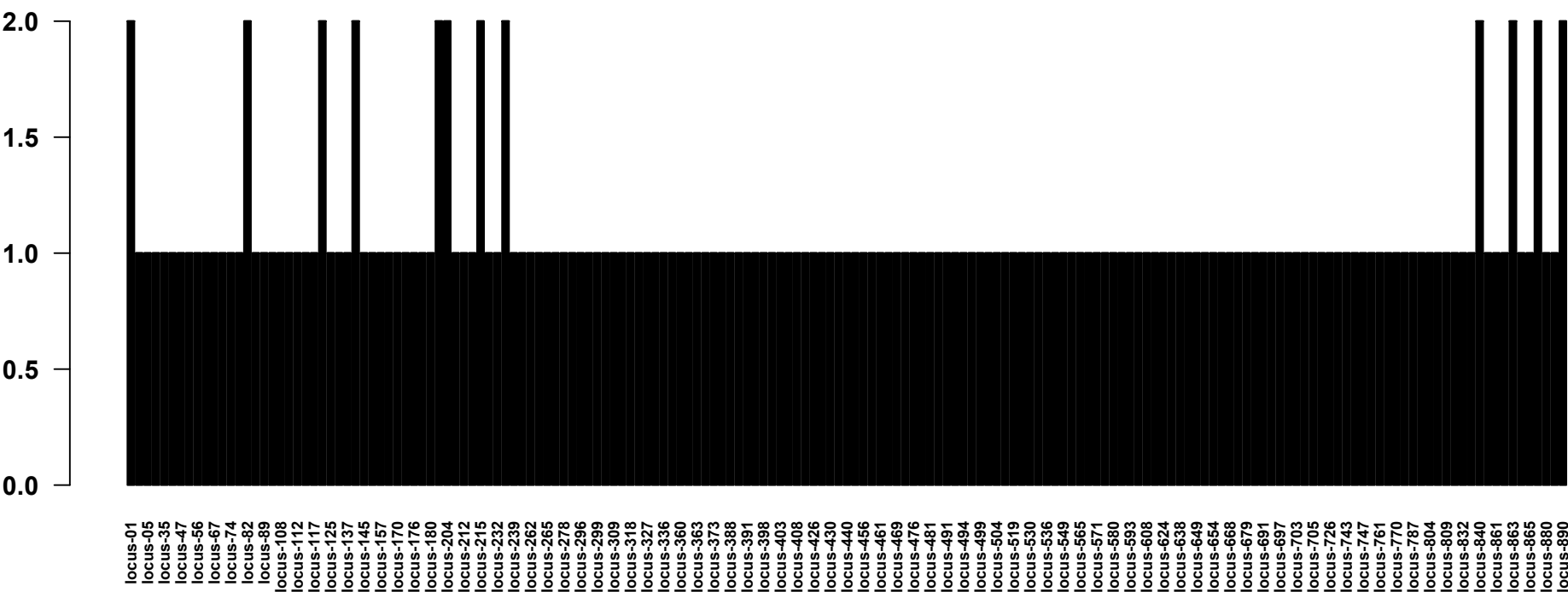

## TIER 2

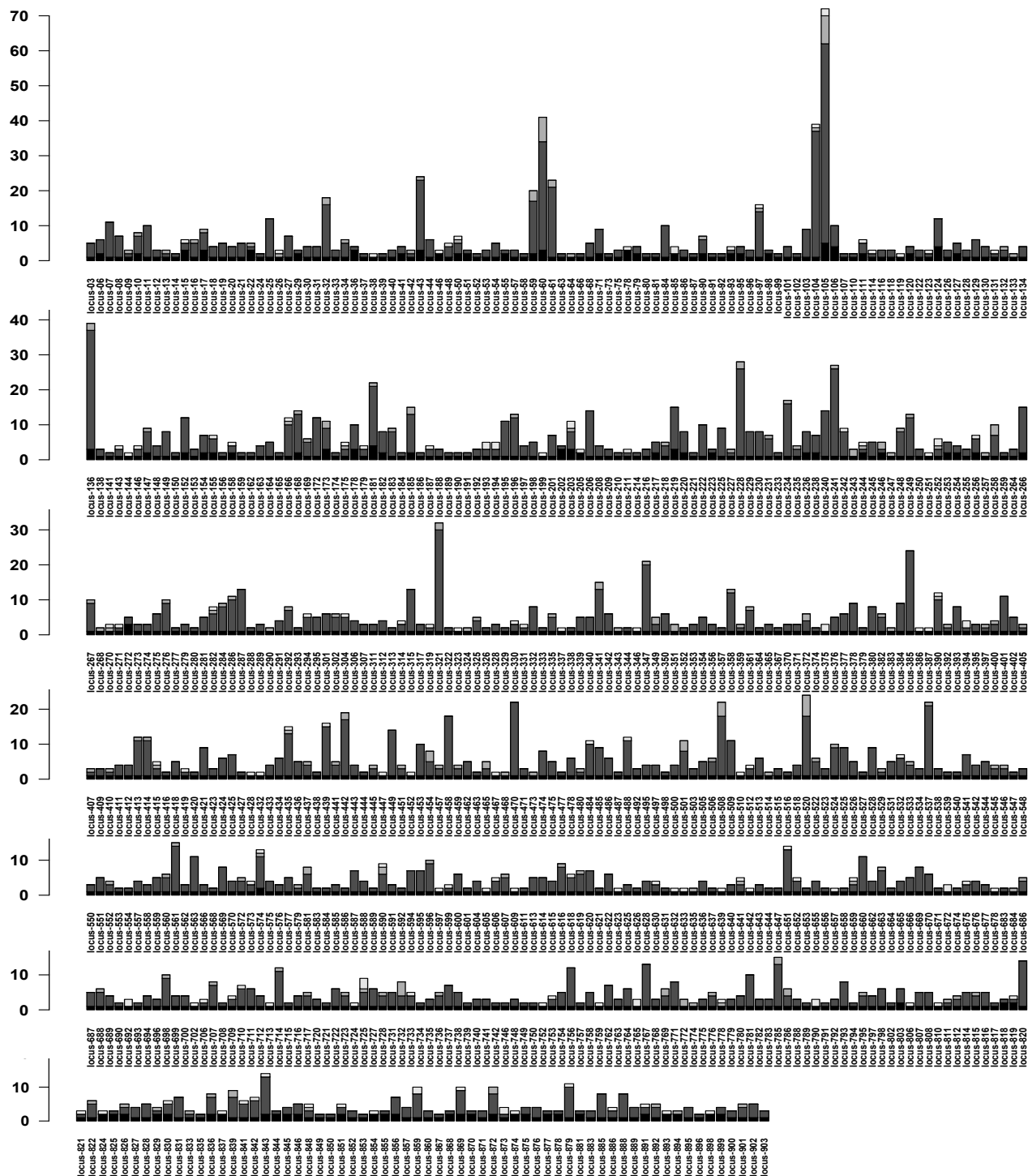

# TIER 3

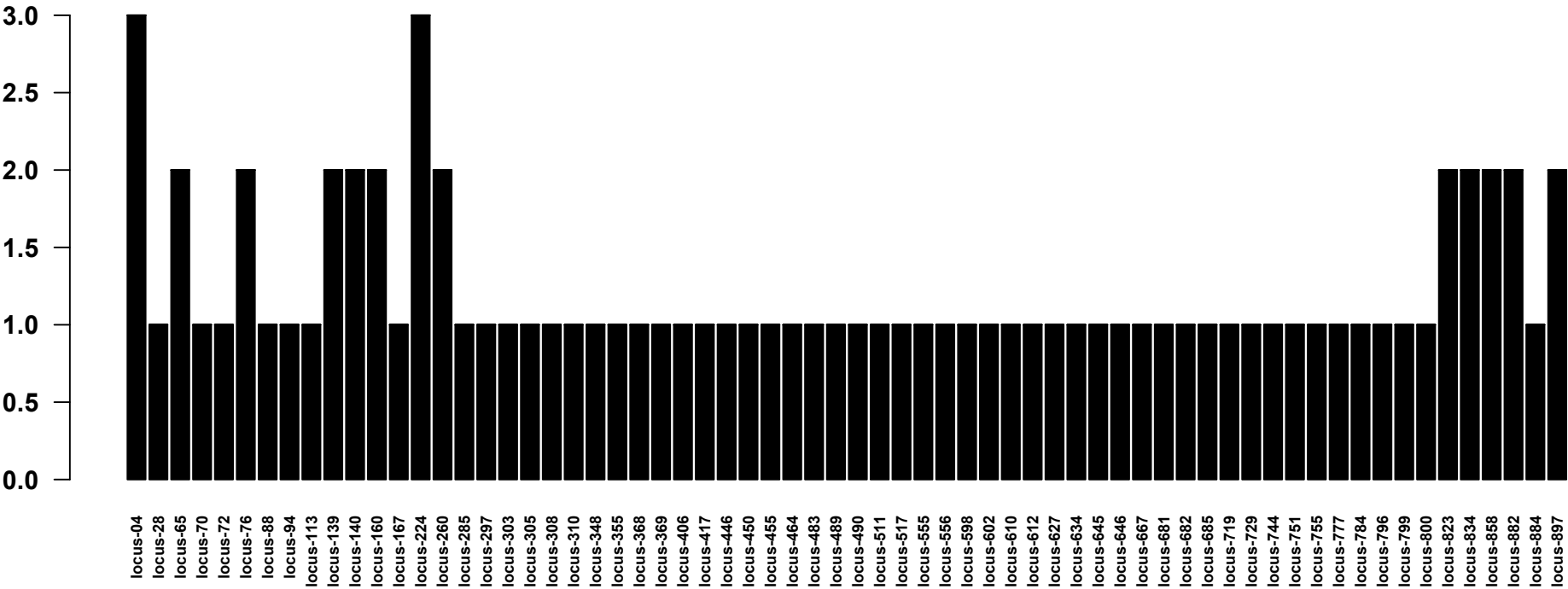

Supplement: S4 File — Bar charts showing the distribution of tier assignments for each of the GWA studies considered. Tier assignments for each of the 903 loci identified in a recent blood pressure GWAS [27]. (PDF) [file pone.0244904.s009.pdf]
