# Supplementary material for: Interpreting coronary artery disease GWAS results: A functional genomics approach assessing biological significance
Source: PLoS One. 2022 Feb 22;17(2):e0244904. doi: 10.1371/journal.pone.0244904 (PMC8863290; doi:10.1371/journal.pone.0244904)
Supplement: S1 File — Gene names corresponding to bar plot presented in Fig 1B. (DOCX) [file pone.0244904.s006.docx]

TEX41

CDKN2B-AS1

TARID

MAPKAPK5-AS1

LINC01405

SBF2-AS1

LINC00954

LINC00310

MIR3936HG

IRF1-AS1

LPAL2

TAF1A-AS1

LINC02659

AC022748.2

AL008729.1

AL137026.1

HOXB-AS1

LINC01312

POM121L9P

AC018742.1

AL139393.2

ZNF259P1

AL139393.1

POC1B-AS1

ADAMTS7P3

AC006435.2

SUMO2P17

LINC02709

AC013565.1

AC007773.1

PSMD8P1

AL592148.3

RPSAP22

AC104083.1

MIR3936

AC116366.2

ANKRD18EP

AL024497.1

AL024497.2

AL109933.2

AC003986.2

AC100802.1

MARCKSL1P1

AP002989.1

AP005018.2

AC025034.1

ATP2B1-AS1

AC126178.1

ADAM1B

RPL21P116

AC006435.3

AC020558.2

AC122129.1

AP000317.1

AC069307.1

GGTLC4P

AL449423.1

LCN1P1

WDR12

PLPP3

ADAMTS7

MRPS6

PLG

ABCG5

CELSR2

MIA3

CALHM2

NBEAL1

ATXN7L2

SARS1

AIDA

MAT2A

SLC22A2

SLC22A4

JCAD

OSR1

HOXB2

MYBPHL

ANKRD10

CTSH

CDK5RAP3

GSTM4

POLR1A

COG5

ABO

TDRD10

SURF1

MFGE8

RGL3

MYO15A

SREBF1

ESYT3

PDLIM4

BCAM

ZNF76

CALCOCO2

USP39

COL4A2

GGCX

NOS3

ABHD2

MAPKAPK5

CHRNB4

KCNH2

KSR2

VAMP5

SYPL2

FAM117B

BCAS3

SNF8

PEMT

DPH1

BROX

MORF4L1

NT5C2

POC1B

TAF1A

AAGAB

TMEM150A

DPY19L3

HDAC9

SMG6

SORT1

ATP5MC1

MFSD13A

ZNF507

COL4A1

PSMA4

LIPA

CYP17A1

PAFAH1B2

ATXN2

ATP2B1

IRF1

LDLR

SGSM2

FES

TMEM116

DRC3

SLC44A2

SLC22A5

ZPR1

TTLL6

FAIM

PCSK9

GFOD1

REXO4

ABCG8

PMAIP1

SAYSD1

CDKN2B

BORCS7

ADTRP

PHACTR1

P4HA2

TOM1L2

RAI1

SWAP70

SFTPB

MRAS

HBP1

ZEB2

ZC3HC1

PSRC1

IL6R

LPL

CXCL12

UBE2Z

ANKS1A

TRIB1

CNNM2

BMPR2

SNRPC

KLHDC10

WBP1L

EML1

BCAP29

SFXN2

HOXB3

EDNRA

SMARCA4

SMAD3

ADORA2A

MED22

CARM1

APOE

ALDH2

TSR1

HAPLN3

RAD50

REST

YIPF2

FAM177B

RNF181

GUCY1A1

SLC22A1

ICA1L

FURIN

NME9

TCP11

TBC1D7

RAPH1

CARF

DUS4L-BCAP29

FLT1

APOC1

ZNF438

GUCY1B1

SRR

TOMM40

APOA1

DNAH8

AC119674.2

ATP5MD

APOB

APOA5

CHRNA5

VAMP8

LPA

PDGFD

SH2B3

TEAD3

TAF11

TWIST1

SHE

ILRUN

TCF21

HHIPL1

RASD1

UHRF1BP1

AS3MT

KCNK5

SLC22A3

KCNE2

NOA1

MC4R

BSND

A4GNT

AC093908.1

SCUBE3

ARL3

GALNT4

SLC5A3

AL359922.1

AC011481.3
