# Supplementary material for: Interpreting coronary artery disease GWAS results: A functional genomics approach assessing biological significance
Source: PLoS One. 2022 Feb 22;17(2):e0244904. doi: 10.1371/journal.pone.0244904 (PMC8863290; doi:10.1371/journal.pone.0244904)

Example of a locus (*LIPA*) implicated by GWAS taken from ensemble.org. There are numerous annotated protein-coding and non-coding transcripts in close proximity and overlapping one another.


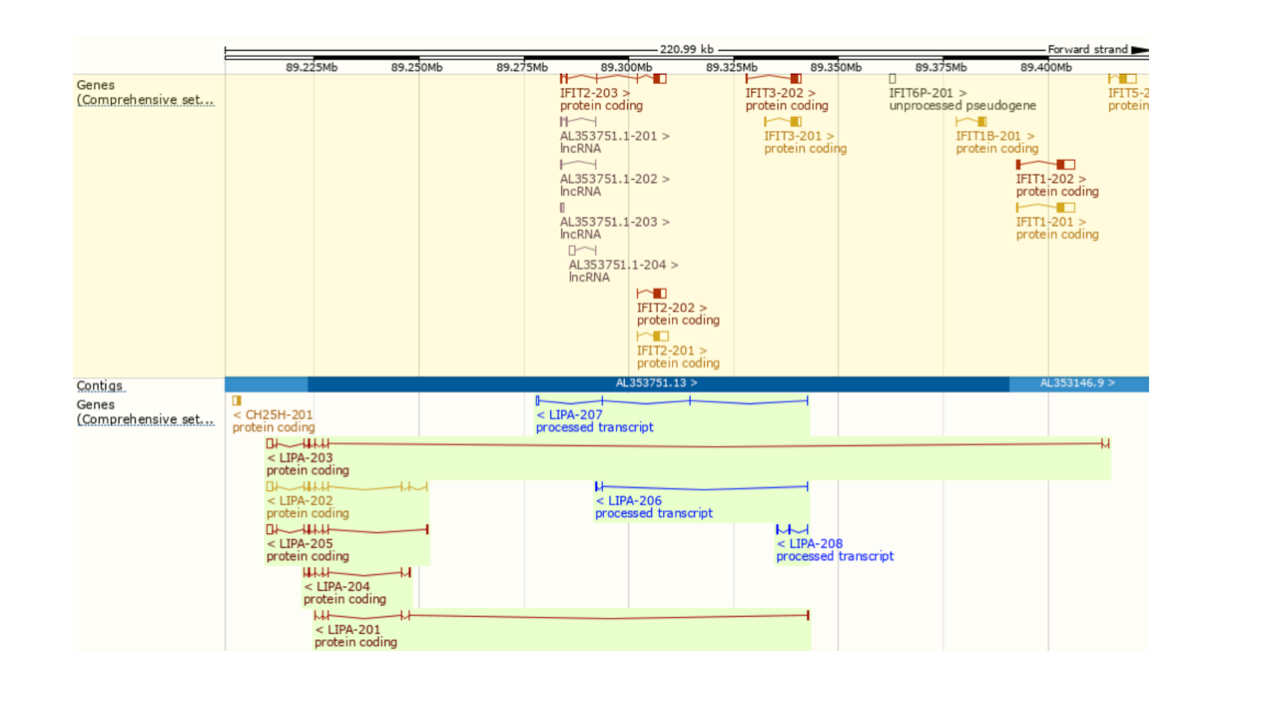

Supplement: S2 File — Example of a locus (LIPA) implicated by GWAS taken from ensemble.org. There are numerous annotated protein-coding and non-coding transcripts in close proximity and overlapping one another. (DOCX) [file pone.0244904.s007.docx]
