# Supplementary material for: Interpreting coronary artery disease GWAS results: A functional genomics approach assessing biological significance
Source: PLoS One. 2022 Feb 22;17(2):e0244904. doi: 10.1371/journal.pone.0244904 (PMC8863290; doi:10.1371/journal.pone.0244904)
Supplement: S3 File — Table of 58 GWAS loci including tier designation, SNPs considered, GWAS annotation, and genes introduced by eQTL, sQTL, and position. Also includes additional text describing Tier 3 loci and the expanded search for candidate genes. (DOCX) [file pone.0244904.s008.docx]

| **Locus** | **Tier** | **SNP** | **LD**  **(SNP \| r2)** | **GWAS Annotation** | **eQTL** | **sQTL** | **Location** |
| --- | --- | --- | --- | --- | --- | --- | --- |
| 01 | 2A | rs11206510 |  | PCSK9 | PCSK9  BSND |  |  |
|  |  |  |  |  |  |  |  |
| 02 | 2A | rs17114036 | rs9970807 \| 0.6 | PLPP3 | PLPP3 |  | AC119674.2 PLPP3 |
|  | 2A | rs9970807 |  | PLPP3 | PLPP3 |  | AC119674.2  PLPP3 |
|  |  |  |  |  |  |  |  |
| 03 | 2A | rs646776 | rs7528419 \| 0.85 | SORT1 | ATXN7L2  CELSR2  GSTM4  PSRC1  SORT1  SYPL2 | MYBPHL |  |
|  | 2A | rs7528419 |  | SORT1 | ATXN7L2  CELSR2  GSTM4  PSRC1  SARS1  SORT1  SYPL2 | MYBPHL | CELSR2 |
|  |  |  |  |  |  |  |  |
| 04 | 2A | rs4845625 | rs6689306 \| 0.82 | IL6R | IL6R  PSMD8P1  TDRD10 | IL6R  SHE  TDRD10 | IL6R |
|  | 2A | rs6689306 |  | IL6R | IL6R  TDRD10 | IL6R  SHE  TDRD10 | IL6R |
|  |  |  |  |  |  |  |  |
| 05 | **2A** | rs17464857 | rs67180937 \| < 0.2 | MIA3 | AL592148.3  MIA3  TAF1A  TAF1A-AS1 |  | TAF1A |
|  | **2A** | rs17465637 | rs67180937 \| 0.98 | MIA3 | AIDA  AL592148.3  BROX  FAM177B  MIA3  TAF1A  TAF1A-AS1 | MIA3  TAF1A-AS1 | MIA3 |
|  | 1 | rs67180937 |  | MIA3 |  |  | MIA3 |
|  |  |  |  |  |  |  |  |
| 06 | 2C | rs16986953 |  | AK097927 | OSR1 |  |  |
|  |  |  |  |  |  |  |  |
| 07* | **2A** | rs515135 | rs34908258 \| 0.51 | APOB | AC018742.1  APOB |  |  |
|  | 3 | rs34908258 |  | APOB |  |  |  |
|  |  |  |  |  |  |  |  |
| 08* | 1 | rs6544713 | \| < 0.2 | ABCG5  ABCG8 | ABCG8 |  | ABCG8 |
|  | 1 | rs538780541 |  | ABCG5  ABCG8 |  |  | ABCG8 |
|  | 1 | rs1004684384 |  | ABCG5  ABCG8 |  |  | ABCG8 |
|  | 1 | rs1191707939 |  | ABCG5  ABCG8 |  |  | ABCG8 |
|  | 1 | rs1249058410 |  | ABCG5  ABCG8 |  |  | ABCG8 |
|  | 1 | rs960463115 |  | ABCG5  ABCG8 |  |  | ABCG8 |
|  | 1 | rs1483199541 |  | ABCG5  ABCG8 |  |  | ABCG8 |
|  |  |  |  |  |  |  |  |
| 09 | 2A | rs1561198 | rs7568458 \| 0.86 | GGCX  VAMP5  VAMP8 | GGCX  MAT2A  RNF181  RPSAP22  TMEM150A  USP39  VAMP5  VAMP8 | GGCX  RNF181  SFTPB  TMEM150A  VAMP5  VAMP8 |  |
|  | 2A | rs7568458 |  | GGCX  VAMP5  VAMP8 | GGCX  MAT2A  POLR1A  RNF181  RPSAP22  TMEM150A  USP39  VAMP5  VAMP8 | GGCX  RNF181  SFTPB  TMEM150A  VAMP5  VAMP8 | GGCX |
|  |  |  |  |  |  |  |  |
| 10 | 2B | rs2252641 | rs17678683 \| <0.2 | ZEB2  AC074093 |  |  | AC074093 |
| 10 | 2B | rs17678683 |  | ZEB2  AC074093 | ZEB2 | ZEB2 | LINC01412 |
|  |  |  |  |  |  |  |  |
| 11* | 2A | rs6725887 | rs201810558 \| 0.98 | WDR12 | BMPR2  CARF  FAM117B  ICA1L  NBEAL1  WDR12 | CARF  RAPH1 | WDR12 |
|  | 2A | rs201810558 |  | WDR12 | BMPR2  CARF  FAM117B  ICA1L  NBEAL1  WDR12 | CARF  RAPH1 | CARF  WDR12 |
|  |  |  |  |  |  |  |  |
| 12* | **2A** | rs9818870 | < 0.2 | MRAS | A4GNT  ESYT3  MRAS  NME9 | FAIM  MRAS | MRAS |
|  | 1 | rs201399015 |  | MRAS |  |  | MRAS |
|  | 1 | rs79208586 |  | MRAS |  |  | MRAS |
|  | 1 | rs1553800573 |  | MRAS |  |  | MRAS |
|  | 1 | rs959582615 |  | MRAS |  |  | MRAS |
|  |  |  |  |  |  |  |  |
| 13 | **2A** | rs1878406 | rs4593108 \| < 0.2 | EDNRA | AC093908.1  EDNRA |  |  |
|  | 1 | rs4593108 |  | EDNRA | EDNRA |  |  |
|  |  |  |  |  |  |  |  |
| 14 | 2A | rs7692387 | rs72689147 \| 0.96 | GUCY1A3 | AC104083.1  GUCY1A3  GUCY1B1 | GUCY1A3 | GUCY1A3 |
|  | 2A | rs72689147 |  | GUCY1A3 | AC104083.1  GUCY1A3  GUCY1B1 | GUCY1A3 | GUCY1A3 |
|  |  |  |  |  |  |  |  |
| 15 | 2A | rs273909 |  | SLC22A4  SLC22A5 | AC116366.2  IRF1-AS1  MIR3936  MIR3936HG  P4HA2  PDLIM4  SLC22A4  SLC22A5 | IRF1  IRF1-AS1  MIR3936HG  P4HA2  RAD50 | MIR3936HG  SLC22A4 |
|  |  |  |  |  |  |  |  |
| 16 | 1 | rs6903956 |  | ADTRP |  | ADTRP | ADTRP |
|  |  |  |  |  |  |  |  |
| 17 | 2A | rs12526453 | rs9349379 \| < 0.2 | PHACTR1 | AL008729.1  PHACTR1 |  | PHACTR1 |
|  | 2A | rs9349379 |  | PHACTR1 | AL008729.1  GFOD1  PHACTR1  TBC1D7 |  | PHACTR1 |
|  |  |  |  |  |  |  |  |
| 18 | 2A | rs17609940 |  | ANKS1A | ANKS1A  SCUBE3  SNRPC  TAF11  TEAD3  UHRF1BP1  ZNF76 | ANKS1A  ILRUN  SNRPC  TCP11 | ANKS1A |
|  |  |  |  |  |  |  |  |
| 19 | 2A | rs10947789 | rs56336142 \| 0.41 | KCNK5 | DNAH8  KCNK5 |  | KCNK5 |
|  | 2A | rs56336142 |  | KCNK5 | ANKRD18EP  KCNK5  SAYSD1 |  |  |
|  |  |  |  |  |  |  |  |
| 20 | 2A | rs12190287 | rs12202017 \| < 0.2 | TCF21 | AL024497.1  AL024497.2  TARID  TCF21 | LINC01312 | TCF21 |
|  | 2A | rs12202017 |  | TCF21 | AL024497.1  AL024497.2  TARID  TCF21 | LINC01312 | LINC01312  TARID |
|  |  |  |  |  |  |  |  |
| 21 | 1 | rs2048327 | rs55730499 \| < 0.2 | LPA  LPAL2  SLC22A3 | LPA  LPAL2  SLC22A3 | LPAL2  SLC22A3 | SLC22A3 |
|  | **2A** | rs3798220 | rs55730499 \| < 0.2 | LPA  LPAL2  SLC22A3 | LPA  SLC22A1 |  | LPA |
|  | 1 | rs55730499 |  | LPA  LPAL2  SLC22A3 | SLC22A3 | SLC22A3 | LPA |
|  |  |  |  |  |  |  |  |
| 22 | 2A | rs4252120 | rs4252185 \| <0.2 | PLG | AL109933.2  AL139393.1  AL139393.2  PLG | SLC22A3 | PLG |
|  | 2A | rs4252185 |  | PLG | SLC22A2  SLC22A3 |  | PLG |
|  |  |  |  |  |  |  |  |
| 23 | **2A** | rs2023938 | rs2107595 \| <0.2 | HDAC9 | AC003986.2 |  | HDAC9 |
|  | 2C | rs2107595 |  | HDAC9 | AC003986.2  TWIST1 |  |  |
|  |  |  |  |  |  |  |  |
| 24 | 2A | rs10953541 |  | 7q22 | BCAP29  HBP1 | COG5  HBP1 | BCAP29  DUS4L-BCAP29 |
|  |  |  |  |  |  |  |  |
| 25 | 2A | rs11556924 |  | ZC3HC1 | KLHDC10  ZC3HC1 | ZC3HC1 | AC073320.2  ZC3HC1 |
|  |  |  |  |  |  |  |  |
| 26 | 2A | rs264 |  | LPL | AC100802.1  LPL |  | LPL |
|  |  |  |  |  |  |  |  |
| 27 | 2C | rs2954029 |  | TRIB1 |  |  | AC091114.1 |
|  |  |  |  |  |  |  |  |
| 28 | 2A | rs3217992 | rs2891168 \| < 0.2 | 9p21 | CDKN2B | AL449423.1  CDKN2B-AS1 | AL359922.1  CDKN2B  CDKN2B-AS1 |
|  | 2A | rs4977574 | rs2891168 \| 0.98 | 9p21 |  | AL449423.1  CDKN2B-AS1 | CDKN2B-AS1 |
|  | 2A | rs2891168 |  | 9p21 |  | AL449423.1  CDKN2B-AS1 | CDKN2B-AS1 |
|  |  |  |  |  |  |  |  |
| 29 | 2A | rs579459 | rs2519093 \| 0.81 | ABO | ABO  SURF1 | ABO  LCN1P1 |  |
|  | 2A | rs2519093 |  | ABO | ABO  MED22  REX04  SURF1 | ABO  LCN1P1 | ABO |
|  |  |  |  |  |  |  |  |
| 30 | **2A** | rs2505083 | rs2487928 \| 0.84 | KIAA1462 | JCAD  ZNF438 | JCAD | JCAD |
|  | 1 | rs2487928 |  | KIAA1462 | JCAD | JCAD | JCAD |
|  |  |  |  |  |  |  |  |
| 31 | 2C^#^ | rs2047009 | rs1870634 \| 0.47 | CXCL12 | LINC02659 |  |  |
|  | 2C^#^ | rs501120 | rs1870634 \| < 0.2 | CXCL12 | AL137026.1 |  |  |
|  | 1^#^ | rs1870634 |  | CXCL12 | CXCL12 |  |  |
|  |  |  |  |  |  |  |  |
| 32 | 1 | rs11203042 | rs1412444 \| < 0.2 | LIPA | LIPA | LIPA | LIPA |
|  | 1 | rs1412444 |  | LIPA | LIPA | LIPA | LIPA |
|  |  |  |  |  |  |  |  |
| 33 | 2B | rs12413409 | rs11191416 \| 0.76 | CNNM2  CYP17A1  NT5C2 | ARL3  AS3MT  BORCS7  CALHM2  CNNM2  CYP17A1-AS1  MARCKSL1P1  MFSD13A  NT5C2  SFXN2  WBP1L | AS3MT  ATP5MD  CYP17A1-AS1  NT5C2  SFXN2  WBP1L | CNNM2 |
|  | 2B | rs11191416 |  | CNNM2  CYP17A1  NT5C2 | ARL3  AS3MT  BORCS7  CALHM2  CNNM2  CYP17A1-AS1  MARCKSL1P1  MFSD13A  NT5C2  SFXN2  WBP1L | AS3MT  ATP5MD  CYP17A1-AS1  NT5C2  SFXN2  WBP1L | PFN1P11 |
|  |  |  |  |  |  |  |  |
| 34 | 2A | rs974819 | rs2128739 \| 0.88 | PDGFD | PDGFD | AP002989.1 | AP002989.1 |
|  | 2A | rs2128739 |  | PDGFD | AP002989.1  PDGFD | AP002989.1 | AP002989.1 |
|  |  |  |  |  |  |  |  |
| 35 | 2C | rs964184 |  | APOA1  APOA5  ZNF259 | AP005018.2  PAFAH1B2  ZPR1 |  | ZPR1 |
|  |  |  |  |  |  |  |  |
| 36 | 2A | rs7136259 | rs2681472 \| < 0.2 | ATP2B1 | AC025034.1  ATP2B1  GALNT4  POC1B-AS1 |  | ATP2B1 |
|  | 2A | rs2681472 |  | ATP2B1 | AC025034.1  AC126178.1  ATP2B1  ATP2B1-AS1  GALNT4  POC1B  POC1B-AS1 |  | ATP2B1 |
|  |  |  |  |  |  |  |  |
| 37 | 2A | rs3184504 |  | SH2B3 | ADAM1B  ALDH2  LINC01405  MAPKAPK5  TMEM116 | LINC01405  MAPKAPK5-AS1 | ATXN2  SH2B3 |
|  |  |  |  |  |  |  |  |
| 38 | 1 | rs9319428 |  | FLT1 | FLT1 |  | FLT1 |
|  |  |  |  |  |  |  |  |
| 39 | 1 | rs4773144 | rs11838776 \| < 0.2 | COL4A1  COL4A2 | COL4A1 |  | COL4A2 |
|  | 1 | rs9515203 | rs11838776 \| < 0.2 | COL4A1  COL4A2 | COL4A1  COL4A2 |  | COL4A2 |
|  | **2A** | rs11838776 |  | COL4A1  COL4A2 | ANKRD10  COL4A1 | COL4A2 | COL4A2 |
|  |  |  |  |  |  |  |  |
| 40 | 1 | rs2895811 | rs10139550 \| 0.42 | HHIPL1 | HHIPL1 |  | HHIPL1 |
|  | **2A** | rs10139550 |  | HHIPL1 | EML1  HHIPL1 |  | HHIPL1 |
|  |  |  |  |  |  |  |  |
| 41 | 2A | rs7173743 | rs4468572 \| 0.52 | ADAMTS7 | AC022748.2  ADAMTS7  ADAMTS7P3  CHRNB4  CHRNA5  CTSH  MORF4L1  PSMA4  RPL21P116 | AC022748.2  ADAMTS7 | MORF4L1 |
|  | 2A | rs4468572 |  | ADAMTS7 | AC022748.2  ADAMTS7 ADAMTS7P3  CHRNA5  CTSH  MORF4L1 | AC022748.2  ADAMTS7  CHRNB4 | MORF4L1 |
|  |  |  |  |  |  |  |  |
| 42 | 1 | rs17514846 |  | FES  FURIN | FES  FURIN | FES | FURIN |
|  |  |  |  |  |  |  |  |
| 43 | 2A | rs216172 |  | SMG6 | AC006435.2  AC006435.3  SGSM2  SRR  TSR1 | DPH1 | SMG6 |
|  |  |  |  |  |  |  |  |
| 44 | 2B | rs12936587 |  | PEMT  RAI1  RASD1 | AC020558.2  AC122129.1  DRC3  MYO15A  PEMT  SREBF1  TOM1L2 | PEMT  RAI1  TOM1L2 |  |
|  |  |  |  |  |  |  |  |
| 45 | 2A | rs46522 |  | UBE2Z | ATP5MC1  CALCOCO2  CDK5RAP3  HOXB-AS1  HOXB2  HOXB3  SNF8  SUMO2P17  TTLL6  UBE2Z | ATP5MC1  CALCOCO2  SNF8  UBE2Z | UBE2Z |
|  |  |  |  |  |  |  |  |
| 46 | 2C | rs1122608 | rs56289821 \| < 0.2 | LDLR | CARM1  SMARCA4  YIPF2 | SMARCA4 | SMARCA4 |
|  | 2C | rs56289821 |  | LDLR | RGL3  SLC44A2  SMARCA4 |  | SMARCA4 |
|  |  |  |  |  |  |  |  |
| 47 | 2C | rs2075650 | rs4420638 \| < 0.2 | APOC1  APOE | BCAM | TOMM40 | TOMM40 |
|  | 2B | rs445925 | rs4420638 \| < 0.2 | APOC1  APOE | APOE |  | AC011481.3 |
|  | **2A** | rs4420638 |  | APOC1  APOE | APOC1  APOE | TOMM40 |  |
|  |  |  |  |  |  |  |  |
| 48 | 2A | rs9982601 | rs28451064 \| 0.4 | KCNE2 | AP000317.1  KCNE2  LINC00310  MRPS6  SLC5A3 |  | AP000317.1  AP000317.2 |
|  | 2A | rs28451064 |  | KCNE2 | AP000317.1  KCNE2  LINC00310  MRPS6  SLC5A3 |  | AP000317.1  AP000317.2 |
|  |  |  |  |  |  |  |  |
| 49 | 2A | rs17087335 |  | NOA1  REST | AC069307.1  REST |  | NOA1 |
|  |  |  |  |  |  |  |  |
| 50 | 2A | rs3918226 |  | NOS3 | KCNH2  NOS3 |  | NOS3 |
|  |  |  |  |  |  |  |  |
| 51 | 2A | rs10840293 |  | SWAP70 | LINC02709  SBF2-AS1  SWAP70 | SBF2-AS1 | SWAP70 |
|  |  |  |  |  |  |  |  |
| 52 | 2A | rs56062135 |  | SMAD3 | AAGAB  SMAD3 | SMAD3 | SMAD3 |
|  |  |  |  |  |  |  |  |
| 53 | 2B | rs8042271 |  | ABHD2  MFGE8 | AC013565.1  HAPLN3  MFGE8 |  |  |
|  |  |  |  |  |  |  |  |
| 54 | 1 | rs7212798 |  | BCAS3 |  |  | BCAS3 |
|  |  |  |  |  |  |  |  |
| 55 | 3 | rs663129 |  | MC4R  PMAIP1 |  |  |  |
|  |  |  |  |  |  |  |  |
| 56 | 2B | rs180803 |  | ADORA2A  POM121L9P | GGTLC4P  POM121L9P |  | BCRP1  POM121L9P |
|  |  |  |  |  |  |  |  |
| 57 | 1 | rs11830157 |  | KSR2 |  |  | KSR2 |
|  |  |  |  |  |  |  |  |
| 58 | 2B | rs12976411 |  | LOC400684  ZNF507 | DPY19L3  ZNF507 | AC007773.1 | AC007773.1 |

* Table 2 of Nikpay et al. (Nikpay *et al.*, 2015) provides a chr:position:I/D notation for these loci, for which the corresponding rs identifier is ambiguous. All rs ids that correspond to an insertion/deletion variant spanning the provided chr:position have been included.

# Locus overall designated as 2A given two SNPs would be classified as tier 2C and the other to tier 1

**BOLD**: For those loci with multiple SNPs and different tier designations depending on the SNP considered, the designation assigned to the locus overall is bolded.
